# The Covid-19 pandemic stress the need to build resilient production ecosystems

**DOI:** 10.1007/s10460-020-10105-w

**Published:** 2020-05-23

**Authors:** Line J. Gordon

**Affiliations:** Stockholm Resilience Centre, Stockholm University, Stockholm, Sweden

I have always loved the word resilience. To me, it is the smell of wet soil and the first green leaves after a long drought. It is hearing my dad tell his memoirs to his grandkids after I thought I would lose him from an illness. Resilience is the useful lessons learnt during difficult times, that can result in a world with more compassion and care.

The current Covid-19 pandemic, and its connections to human health, the biosphere on which we depend, and our food systems, show the need to embrace resilience; to live with uncertainty, prepare for disturbances, learn and experiment; to act with empathy and solidarity; and to imagine a better world.

## What is resilience?

Resilience as defined by sustainability scientists is to maintain development in the face of both surprising and expected change, when thresholds between alternative pathways exists of which some may be less desirable than others, and where it is difficult or even impossible to turn back once the threshold is crossed (Folke 2006).

For example, a heatwave over a coral reef with low resilience, can result in algae taking over the corals. This often cause species loss, result in lower fish abundance, and large losses to the tourism sector. The algae are hard or impossible to get rid of, the corals might never come back. The people living off the reef can lose livelihoods dependent on tourism and fisheries. A resilient reef would be able to return back and the communities around it continue to develop.

## To value what sustains us in crisis, that we normally take for granted

When a crisis hit, the resilience often comes from places less anticipated or even neglected. For example, a case study of a shift from algae to coral dominated communities on the Great Barrier Reef showed that recovery was primarily driven by one single species that is relatively rare on the reef, a species that easily can go extinct without notice under normal conditions but that is needed in crisis (Bellwood et al. 2006). Similarly, it is the less dominating species in a mature forest that often invades first and prepare the grounds for the return of the forest after a fire. Species that are less visible, and seem less valuable, can have a big role to play for the system to rebuild itself.

During the Covid-19 pandemic, we see this in the realization of the immensely important work done not just by doctors, but also by workers caring for our elders. In Sweden, undervaluing their work for a long time in terms of allowing a high degree of job insecurity and low pay seem to be one of the major drivers behind the relatively high death tolls we now face in the country as the virus spread in old age homes. In the food system we suddenly realize our dependence on migrant farm workers who cannot pass across borders thus putting this year’s harvest at risk, and on the people working with food distribution, who can step up and help eat-in restaurants turn to home delivery services, and get groceries to our door as we avoid stores.

## It is time to rebuild the resilience of the global food system

As a human society we have rapidly transformed this planet. Much of the biosphere’s resources have been appropriated for the production of harvestable biomass in the form of food, fuel and fibre. We have created a global production ecosystem that is homogenous, highly connected and characterized by weakened internal feedbacks. We get high and more predictable yields of biomass in the short term, but with new conditions for novel and pervasive risks to emerge in the longer term (Nyström et al. 2019). We have too little understanding today of what we already have lost of the invisible species and social-ecological connections that build the resilience of the global production ecosystem and will sustain us in the future and we must urgently step up this type of research.

We are now in the midst of what is arguably the largest global crisis our generation has faced. My sincere hope is that the Covid-19 pandemic can help us recognize the invisible support systems we depend on to ensure that we nurture them as we continue our development into the future.
